# Relationship between sleep–wake patterns, occupational stress and sickness absence among nurses

**DOI:** 10.3389/fpsyg.2026.1935070

**Published:** 2026-08-28

**Authors:** Katarzyna Lisowicz, Katarzyna Tomaszewska, Dawid Makowicz, Marek Sobolewski, Krystyna Kowalczuk

**Affiliations:** 1 Institute of Health State University of Applied Sciences in Krosno, Krosno, Poland; 2 Faculty of Health Care, Rev. Bronisław Markiewicz State University of Applied Sciences in Jarosław, Jarosław, Poland; 3 Faculty of Management, Rzeszów University of Technology, Rzeszów, Poland; 4 Department of Integrated Medical Care, Medical University of Białystok, Białystok, Poland

**Keywords:** nurses, occupational stress, sickness absence, sleep–wake characteristics, workload

## Abstract

Individual sleep–wake characteristics play an important role in daily functioning and adaptation to shift work. They are associated with alertness, sleep quality, cognitive performance, concentration, and the ability to cope with occupational demands. High levels of occupational stress and chronic fatigue have also been identified as factors associated with sickness absence among nursing staff.

**Objective:**

The aim of the study was to assess the relationship between sleep–wake characteristics, occupational stress, and the use of sick leave in a group of practicing nurses.

**Participants and methods:**

The study included 588 male and female nurses employed in three hospitals in the Podkarpackie Voivodeship. A diagnostic survey method was employed, utilizing the Subjective Work Assessment Questionnaire (SWAQ), the Sleep-Wake Pattern Assessment Questionnaire (SWPAQ), and a proprietary questionnaire covering socio-demographic and work-related data. Statistical analysis was performed using non-parametric tests and linear regression.

**Results:**

The SWPAQ scores were generally centered around the midpoint of the scale, indicating no clear predominance of any particular self-reported sleep–wake characteristic.The analysis revealed statistically significant differences in the use of sick leave depending on age (p = 0.0072), marital status (*p* = 0.0038), having a second job (*p* = 0.0093), and working overtime (p = 0.0048). As Anytime Wakeability increased, the level of perceived stress decreased. The study found that being male was an independent predictor of higher stress levels. In the multivariate model, men scored on average 16.2 points higher on the stress scale than women. However, it is worth emphasizing that the observed relationship should not be interpreted as the effect of gender alone, but rather as a result of interactions between many coexisting factors, including psychological conditions.

**Conclusions:**

Higher levels of work-related stress were associated with a greater tendency to fall asleep at any time of the day, and with a reduced ability to stay awake. Independent factors associated with higher stress levels included: being male, working a two-shift system, and working overtime. Higher levels of work-related stress were significantly associated with more frequent use of sick leave. Furthermore, a higher frequency of taking sick leave was associated with older age, higher levels of work-related stress, and taking on additional work.

## 1 Introduction

The circadian rhythm plays an important role in regulating physiological, cognitive, and behavioral processes in humans. Its proper functioning determines, amongst other things, the quality of sleep, the level of alertness, the ability to concentrate, and the ability to adapt to environmental stressors. Nurses performing shift work, especially those with night shifts, are particularly vulnerable to circadian rhythm disruptions (Woodhead et al., 2016; Woo et al., 2020). Shift work leads to a discrepancy between the body's biological rhythm and the demands of the work environment, which can result in sleep disturbances, chronic fatigue, reduced mental and physical performance, and a decline in mental wellbeing. The literature also highlights the link between circadian rhythm disorders, occupational stress, and burnout among medical staff (World Health Organization, 2019; Wudarczyk et al., 2025).

High levels of work-related stress and chronic fatigue are also recognized as factors contributing to sickness absence amongst nursing staff. This phenomenon has an impact both on the employee performance and the organization of healthcare, affecting staff availability and continuity of patient care. Prolonged exposure to stressors can also lead to occupational burnout, which according to the ICD-11 classification is associated with chronic workplace stress and adversely affects the performance of all healthcare professionals, especially nurses (World Health Organization, 2019).

The evidence from scientific research shows that shift work, including night shifts, has adverse psychological and physiological effects, primarily disrupting the normal circadian rhythm, reducing sleep efficiency in the morning, causing health problems (hormonal distress), and reducing work performance during night shifts (Wudarczyk et al., 2025). Job dissatisfaction is also common, leading to social isolation, anger, and frustration. Dysregulation of nurses' circadian rhythms can result in changes in wakefulness and sleep patterns, and the ability to respond quickly in emergencies, such as life-threatening conditions. Long-term reluctance to work, fatigue and stress have a negative impact on nurses' wellbeing, ultimately increasing the chance of taking sick leave (Kohnen et al., 2026; Rahnfeld et al., nd). This is a very common phenomenon nowadays. A report published by the Supreme Council of Nurses and Midwives shows that the average age of nurses is 55, and consequently, nurses are exhausted by many years of practicing their profession, often working night shifts. Disruption to the circadian rhythm has a stressful impact on the body. Higher stress levels among nurses can also lead to taking sick leave. This phenomenon is primarily caused by significant responsibility for patients, staff shortages and work overload, night work and resultant sleep disorders, exposure to pain, suffering, and death, and being with patients during their most difficult moments (Bakker and Demerouti, 2017). What is also important, in addition to the above mentioned factors, is emotional strain and lack of psychological support for healthcare staff. Prolonged stressful situations can lead to anxiety symptoms and disorders, emotional exhaustion, a lack of motivation to work, and difficulties getting up in the morning and concentrating. International studies have confirmed that stress has a negative impact and is one of the major contributors to occupational burnout amongst nurses (Dall'Ora et al., 2020).

Previous research focused on the relationships between shift work, sleep quality, and burnout among nurses. Links between individual sleep–wake characteristics and perceived occupational stress and sickness absence were analyzed less frequently. A better understanding of these relationships may help improve work time management and mental health prophylaxis, as well as reduce negative consequences of shift work in the nursing profession (Bakker and Demerouti, 2017).

The aim of this study was to assess the relationship between self-reported sleep–wake characteristics, occupational stress, and the use of sick leave in a group of practicing nurses.

Specific objectives were:

1. To describe self-reported sleep–wake characteristics and occupational stress among practicing nurses.
2. To examine the associations between individual self-reported sleep–wake characteristics (SWPAQ dimensions) and occupational stress.
3. To assess the relationship between occupational stress and sickness absence.
4. To identify socio-demographic and occupational factors independently associated with occupational stress.
5. To identify socio-demographic, occupational, and work-related factors independently associated with sickness absence.

## 2 Materials and methods

### 2.1 Design of the study

A cross-sectional observational study was conducted using a diagnostic survey method. The study aimed to examine the relationships between self-reported sleep–wake characteristics, occupational stress, and sickness absence among practicing nurses. The study was quantitative in nature and was based on data collected using three instruments: the Sleep–Wake Pattern Assessment Questionnaire (SWPAQ), a multidimensional questionnaire designed to assess individual sleep–wake characteristics; the Subjective Work Assessment Questionnaire (SWAQ); and a proprietary questionnaire covering socio-demographic and occupational characteristics. The study was conducted in accordance with the Strengthening the Reporting of Observational Studies in Epidemiology (STROBE) guidelines for observational studies (von Elm et al., 2007).

### 2.2 Place of the study

The study was conducted in three hospitals located in the Podkarpackie Voivodeship between December 2025 and February 2026. The facilities were selected intentionally, based on their management's consent to the planned research. Each facility was provided with questionnaires, which were distributed among nurses working in wards and outpatient clinics of the healthcare facility participating in the study. The selection of healthcare facilities was based on the diversity of services provided, in order to increase validity of the research.

### 2.3 Study participants

The study was planned to include approximately 200 nurses from each of the three participating hospitals, resulting in an intended sample of 600 participants. Ultimately, 588 fully completed questionnaires were included in the analysis. Thanks to the participants' commitment to the task, 98 % of the intended number of questionnaires were collected. The inclusion criteria were as follows: employment as a nurse, active practice in the profession, and an informed and voluntary willingness to take part in the study. Participants were excluded if they had taken a break from practicing their profession for reasons other than sick leave, or if they refused to take part in the study. It was possible to withdraw from the study at any time. The sample size was calculated to ensure that the estimation of the primary outcome-the proportion of nurses taking sick leave-would have a margin of error of ±4% at the 95% confidece level. The required sample size was estimated to be 600 participants; therefore, the obtained sample of 588 nurses was considered sufficient to acheive the planned estimation precision.

### 2.4 Research tools

A proprietary questionnaire was used to collect socio-demographic and occupational data, including gender, age, place of residence, marital status, educational level, years of professional experience, years of employment at the current workplace, work schedule, overtime work, additional employment, patient-to-nurse ratio during a work shift, and sickness absence. Additional employment was defined as any paid employment undertaken in addition to the participant's primary workplace and was recorded as a dichotomous variable (yes/no). Overtime work was assessed using four response categories (never, occasionally, often, and very often) and was dichotomized for the statistical analyses into no overtime (never) and overtime work (occasionally, often, or very often). Sickness absence was assessed by asking participants to indicate the total duration of sickness absence during the previous 12 months using one of four response categories: no sickness absence, 1–7 days, 8–30 days, or more than 30 days. For the purposes of the logistic regression analysis, this variable was dichotomized into no sickness absence and sickness absence (1–7 days, 8–30 days, or more than 30 days). The above-mentioned variables were included in the statistical analyses examining associations between occupational factors, self-reported sleep–wake characteristics, occupational stress, and sickness absence.

The **Sleep–Wake Pattern Assessment Questionnaire (SWPAQ)** was used to assess individual sleep–wake characteristics. The questionnaire evaluates individual sleep–wake patterns, including morningness–eveningness preferences and subjective sleepability and wakeability. It consists of 72 items grouped into six dimensions: Morningness (M), Eveningness (E), Daytime Wakeability (V), Anytime Wakeability (W), Nighttime Sleepability (S), and Anytime Sleepability (F).

The SWPAQ has demonstrated satisfactory psychometric properties and has been widely used as a standardized instrument in scientific research. In the validation study by Putilov et al. (2017), Cronbach's alpha coefficients were 0.89 for Morningness (M), 0.89 for Eveningness (E), 0.82 for Anytime Wakeability (W), 0.81 for Anytime Sleepability (F), and 0.88 for Nighttime Sleepability (S), indicating good internal consistency of the questionnaire.

In the present study, the Polish version of the Sleep–Wake Pattern Assessment Questionnaire, developed by Jankowski (2008) on the basis of the original instrument by Putilov et al., was used. The Polish adaptation assesses individual sleep–wake characteristics and was described in a chapter devoted to the multidimensional assessment of individual differences in the biological clock.

Occupational stress was assessed using the **Subjective Work Assessment Questionnaire (SWAQ; also referred to as the SWA Questionnaire)**. This standardized instrument evaluates psychosocial workload by assessing employees' subjective perceptions of different aspects of their work. It consists of 55 items covering various work-related demands and psychosocial hazards encountered in the workplace. Responses are rated on a five-point Likert scale, where **1** indicates no or minimal burden and **5** indicates a very high level of perceived burden, stress, aversion, or anxiety associated with a given work-related factor. The total score reflects the overall level of perceived occupational stress. The questionnaire has demonstrated satisfactory psychometric properties, with a Cronbach's alpha coefficient of 0.84 reported for the overall scale. The SWAQ has been widely used in studies conducted in medicine, health sciences, and psychology.

### 2.5 Data collection procedure

Paper questionnaires were distributed to nurses by designated members of the research team in cooperation with nursing managers in participating hospitals. The questionnaires were completed individually, outside the presence of supervisors, during work breaks or after working h, according to participants' preferences. Participation was voluntary and anonymous. No personal identifiers were collected. After completion, questionnaires were placed by participants into sealed collection boxes located in designated areas of each hospital and were subsequently collected by the research team. Hospital managers and supervisors had no access to individual questionnaires or participants' responses. Before participation, all nurses received written information regarding the purpose of the study, confidentiality of the collected data, voluntary participation, and the right to withdraw from the study at any stage without providing a reason.

### 2.6 Statistical analysis

Statistical analysis was conducted with Statistica version 13. The level of statistical significance was set at p < 0.05. In the first stage of the data management, descriptive statistics were calculated for the studied variables, including the arithmetic mean (M), median (Me), standard deviation (SD), minimum and maximum values, and quartiles. To compare occupational stress between 2 independent groups (e.g., gender, additional workplace, place of residence), the Mann–Whitney test was used as a nonparametric counterpart to the *t*-test for independent samples. For the analyses that compared more than two study groups (defined by e.g., marital status, education level, place of employment, work system), the Kruskal–Wallis test was used. Non-parametric tests were employed because the assumptions of normality of distribution and homogeneity of variance were not met. Spearman's rank correlation coefficient (r_s_) was used to assess the relationship between the level of work-related stress and continuous variables, such as age, total length of service and length of service at the current place of employment. This method allows for the assessment of the strength and direction of the monotonic relationship between the analyzed variables and is robust to the presence of outliers.Candidate predictors included the six SWPAQ dimensions, socio-demographic characteristics (sex, age, marital status, education), and occupational variables (place of work, work system, overtime work, additional employment, length of service and other work-related characteristics collected in the questionnaire). In order to identify independent predictors of the overall measure of work-related stress, a multivariate linear regression model was used (Dudek et al., 1999; Sokołowski, 2010; Francuz and Mackiewicz, 2007). The optimal regression model, containing only statistically significant explanatory variables, was selected using a stepwise regression procedure, including both forward and backward selection methods. Prior to performing this analysis, the fulfillment of the model's basic assumptions was assessed, including the linearity of the relationship, the normality of the residual distribution, and the absence of significant multicollinearity between the independent variables. No significant violations of the regression assumptions were found. Only complete questionnaires were included in this model; therefore, the analysis did not include questionnaires with missing data.

### 2.7 Ethical approval

The study was conducted in accordance with the principles of the Declaration of Helsinki. Approval was obtained from the PANS Bioethics Committee in Krosno: Resolution No. 16/2025.

## 3 Results

### 3.1 Study sample characteristics

This article presents selected characteristics of the respondents, which are relevant to the analyzed relationships between self-reported sleep–wake characteristics and occupational stress. The study included 588 nurses. The mean age of the respondents was 43.1 years (*SD* = 11.5), and the mean total length of service was 19.1 years (*SD* = 12.1). On average, each nurse saw 15.1 patients per shift (*SD* = 10.6), with a median of 13 patients. For 50% of the respondents, the patient-to-nurse ratio was between 8 and 18, and the whole range of observed values was from 1 to 80 (Table 1A).

**Table 1A:** Characteristics of the study sample in terms of age, length of service and patient-to-nurse ratio on duty.

Characteristics	*M*	Me	SD	Q1	Q3	Min	Max
Age	43.1	43	11.5	33	53	22	65
Length of service at current position	14.4	10.0	11.8	4.0	23.0	0.5	43
Length of service in total	19.1	18.0	12.1	8.0	30.0	0.5	44
Patient-to-nurse ratio on duty	15.1	13.0	10.6	8.0	18.0	1	80

M, arithmetic mean; Me, median; SD, standard deviation; Q1 – lower quartile; Q3 – upper quartile.

**Table 1B:** Characteristics of the study sample - nominal variables.

Features	Variant	*N* ^a^	%
Sex	Female	489	83.2
	Man	99	16.8
Place of residence	Country	298	50.7
	City	290	49.3
Marital status	Single	91	15.5
	In a relationship (married Or partnered)	424	72.1
	Relationship breakdown	73	12.4
Education	Secondary nursing education	85	14.5
	Bachleor's degree	211	35.9
	Master's degree	291	49.6
Place of	Medical ward	218	37.1
employment	Surgical ward	182	31.0
	Intensive Care Unit	94	16.0
	Primary Health Care	94	16.0
Type of	Full – time employment	522	88.9
employment	Part- time employment	16	2.7
	Contract/B2B	28	4.8
	Civil law contract	21	3.6
Work schedule	One – shift system	134	22.8
	Two – shift system	353	60.1
	Three – shift system	100	17.0
Average weekly	< 40	198	33.8
working hours	40-59	336	57.4
	60-79	40	6.8
	80 and more	11	1.9
Additional	YES	200	34.3
workplace	NO	383	65.7
Overtime work	NO	240	41.0
	YES	345	59.0
Sick leave during	YES	226	38.4
the last year	NO	362	61.6

^*a*^A small number of missing responses were observed for some variables (no more than five cases). Missing data were excluded from the calculation of percentage distributions.

The majority of the study group were women. A typical respondent was married, lived in a rural area or a small town, and most often held a master's or bachelor's degree. The most commonly indicated place of employment was a medical or surgical ward, and the primary form of employment was a full-time employment contract. Most of the respondents worked in a two-shift system, usually for 40–59 h per week. A second job was reported by a lower proportion of the respondents, and overtime was occasional. Among the respondents taking sick leave (n = 229), periods of sick leaves lasting between 1 and 7 days were the most common, accounting for 57.2% of all cases of sickness absence. Sick leaves lasting between 8 and 30 days were taken by 32.3% of those on sick leaves, whilst absences exceeding 30 days occurred in 10.5% of the respondents.

### 3.2 Characteristics of the SWPAQ–sleep–wake characteristics and work-related stress

In the next stage of the analysis, the results obtained with the research tools were described. Distribution of the results for the individual dimensions of the circadian rhythm, as measured using the SWPAQ, and for the level of work-related stress, as measured with the SWAQ, was assessed. The results were presented as arithmetic mean (M), median (Me), standard deviation (SD), lower quartile (Q1), upper quartile (Q3) and minimum and maximum values (Table 2).

**Table 2:** Characteristics of the results obtained with SWPAQ and SWAQ questionnaires.

Variable	*M*	Me	*SD*	Q1	Q3	Min	Max
SWPAQ – circadian rhythm							
Eveningness (E)	−1.3	−12	4.6	−14	2	−112	10
Morningness (M)	0.1	0	4.9	−14	4	−112	12
Anytime wakeability (W)	0.0	0	4.8	−14	4	−112	12
Daytime wakeability (V)	−10.1	0	4.2	−12	2	−112	10
Anytime sleepability (F)	−11.4	0	4.8	−14	2	−112	12
Nighttime sleepability (S)	−10.4	0	4.9	−14	2	−112	12
SWAQ – work-related stress							
Overall stress	146.4	138.0	49.0	107.0	185.5	62	260
Mental workload	23.6	22.0	8.9	16.0	31.0	9	43
Lack of reward	21.2	20.5	7.9	15.0	27.0	8	39
Organizational uncertainty	19.5	18.0	6.9	14.0	25.0	7	35
Social interaction	13.3	12.0	4.8	9.0	17.0	5	25
Sense of threat	13.8	13.0	4.8	10.0	18.0	5	25
Physical strain	10.4	10.0	5.5	4.0	16.0	4	20
Unpleasant working conditions	7.9	9.0	3.7	6.0	10.5	3	15
Lack of control	10.5	9.0	3.9	7.0	14.0	4	20
Lack of support	7.2	7.0	3.2	4.0	9.0	3	15
Sense of responsibility	11.0	10.0	3.9	8.0	14.0	4	

M – arithmetic mean; Me – median; SD – standard deviation; Q1 – lower quartile; Q3 – upper quartile.

The SWPAQ scores were generally centered around the midpoint of the scale, indicating no clear predominance of any particular sleep–wake characteristic in the study group.Slightly lower mean scores were recorded for Eveningness (*M* = −1.3) and Anytime Sleepability (*M* = −1.4). In the SWAQ, the mean score for overall stress was 146.4 (SD = 49.0), with the median of 138, indicating a considerable variation in the level of work-related stress within the study group.

### 3.3 . Correlations between self-reported sleep–wake characteristics and work-related stress

Correlations between self-reported sleep–wake characteristics (as measured by the SWPAQ) and work-related stress (as measured by the SWAQ) were examined. Spearman's rank correlation coefficient was used for the analysis. The results are presented in details in the correlation table. No statistically significant correlations were found between the measures of stress and the measures of Eveningness and Nighttime Sleepability. However, an increase in the stress measures was statistically significantly associated with an increase in Morningness and Anytime Sleepability, as well as a decrease in Anytime Wakeability and Daytime Wakeability. All of the observed correlations showed a small effect size (|rS| < 0.30), despite achieving statistical significance resulting from the large size of the study sample (Table 3).

**Table 3:** Correlations between self-reported sleep–wake and work-related stress.

Subjective work assessment	SWPAQ					
	Eveningness (E)	Morningness (M)	Anytime wakeability (W)	Daytime wakeability (V)	Anytime sleepability (F)	Nighttime sleepability (S)
Overall stress	−0.04 (0.3643)	0.17 (0.0000^***^)	−0.13 (0.0010^**^)	−0.21 (0.0000^***^)	0.21 (0.0000^***^)	−0.01 (0.7941)
Mental workload	−0.04 (0.3178)	0.15 (0.0002^***^)	−0.13 (0.0012^**^)	−0.22 (0.0000^***^)	0.21 (0.0000^***^)	−0.02 (0.7021)
Lack of reward	−0.04 (0.3679)	0.12 (0.0030^**^)	−0.10 (0.0131^*^)	−0.20 (0.0000^***^)	0.21 (0.0000^***^)	−0.01 (0.8873)
Organizational uncertainty	−0.08 (0.0523)	0.18 (0.0000^***^)	−0.14 (0.0005^***^)	−0.21 (0.0004^***^)	0.15 (0.0004^***^)	−0.05 (0.2247)
Social interaction	0.00 (0.9595)	0.15 (0.0002^***^)	−0.11 (0.0088^**^)	−0.16 (0.0001^***^)	0.22 (0.0000^***^)	−0.03 (0.4141)
Sense of threat	−0.02 (0.6038)	0.14 (0.0005^***^)	−0.10 (0.0157^*^)	−0.17 (0.0000^***^)	0.15 (0.0002^***^)	0.02 (0.6201)
Physical strain	−0.04 (0.3911)	0.14 (0.0010^**^)	−0.12 (0.0048^**^)	−0.15 (0.0003^***^)	0.15 (0.0004^***^)	−0.02 (0.6725)
Unpleasant working conditions	0.02 (0.5530)	0.19 (0.0000^***^)	−0.15 (0.0002^***^)	−0.20 (0.0000^***^)	0.12 (0.0042^**^)	0.05 (0.2121)
Lack of control	−0.06 (0.1566)	0.11 (0.0078^**^)	−0.09 (0.0385^*^)	−0.14 (0.0005^***^)	0.19 (0.0000^***^)	0.03 (0.4978)
Lack of support	0.01 (0.7331)	0.05 (0.2617)	−0.07 (0.0718)	−0.12 (0.0025^**^)	0.30 (0.0000^***^)	−0.01 (0.8426)
Sense of responsibility	−0.05 (0.2529)	0.14 (0.0006^***^)	−0.13 (0.0019^**^)	−0.19 (0.0000^***^)	0.15 (0.0002^***^)	−0.02 (0.6934)

^*^, ^**^, ^***^ *p*-values indicate the level of statistical significance.

**Figure 1:**
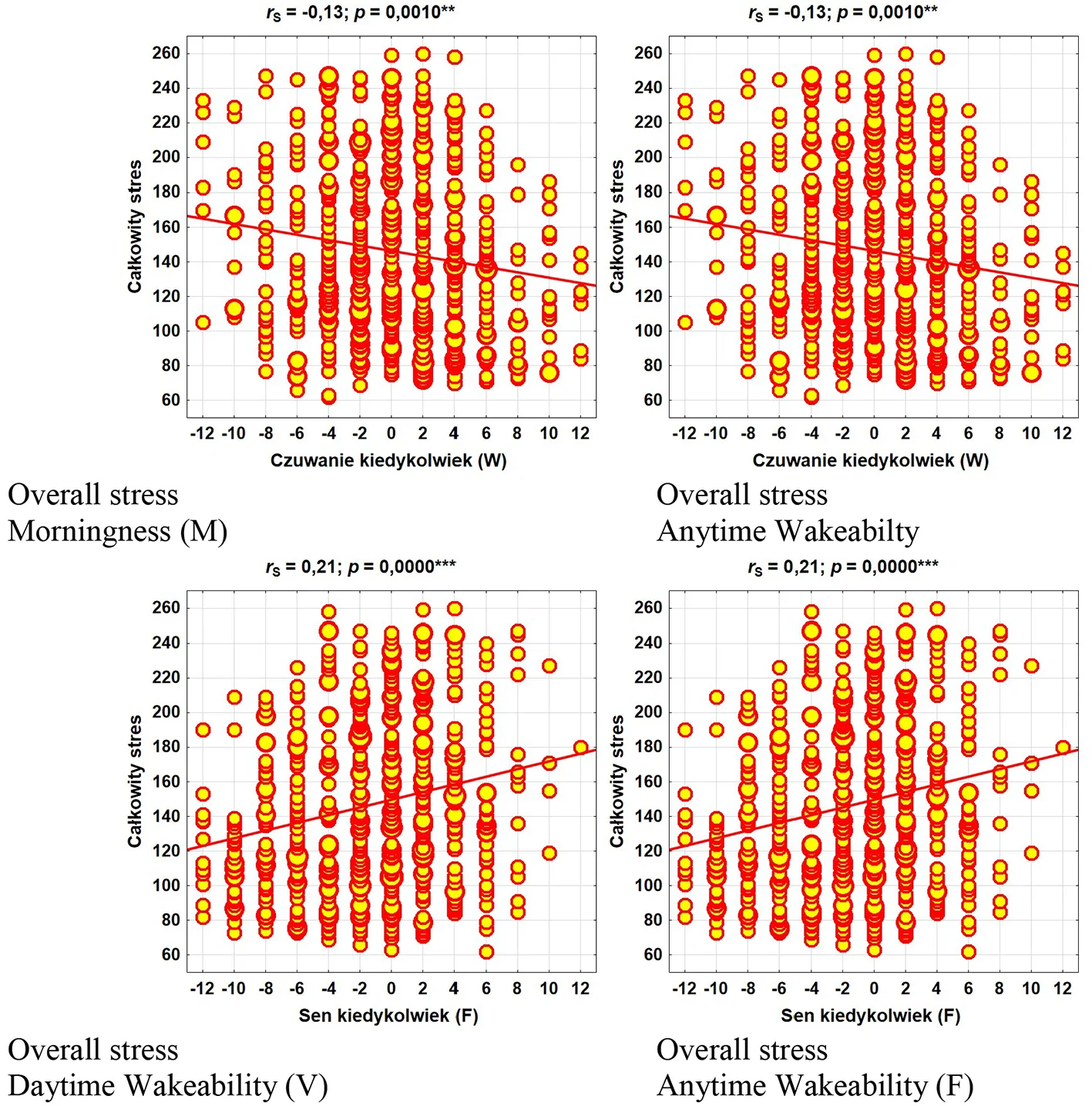
Correlations between the SWPAQ measures and the overall stress measure.

Statistical analysis has shown that the correlations between the SWPAQ measures and the specific stress measures were similar to the correlations with the overall stress measure; therefore, it was these correlations that were subjected to a more detailed graphical analysis. The scatter plots illustrate statistically significant relationships between the four SWPAQ measures and the overall stress measure (Figure 1).

A multivariate analysis was carried out for the total stress measure, in order to verify whether the correlation between the circadian rhythm measures was not merely spurious, resulting from the influence of demographic and occupational factors affecting both the circadian rhythm and stress. The analysis included demographic and occupational characteristics presented in Tables1A, B that showed statistically significant associations with the overall level of work-related stress, as well as all SWPAQ measures. As described in the Methods section, the optimal regression model, presented in Table 4, was identified using both forward and backward stepwise regression procedures. Based on the results obtained, it can be concluded that the significance and direction of the effect of the three SWPAQ measures on the overall stress level have been confirmed. Morningness (M) was the only measure not included in the identified model, which may be due to the fact that the model included factors related to both stress and this SWPAQ measure (such as work system or overtime work).

**Table 4:** Analysis of the impact of selected factors on the overall stress level.

Independent factors	Overall stress *R*^2^ = 16.6% *F* = 16.3 *p* = 0.0000^***^		
	*B* (95% c.i.)	*p*	*ß*
Anytime Wakeability (W)	−11.5 (−12.3; −10.7)	0.0005^***^	−10.15
Daytime Wakeability (V)	−11.5 (−12.4; −10.5)	0.0028^**^	−10.13
Anytime Sleepabilty (F)	2.0 (1.2; 2.8)	0.0000^***^	0.20
Gender (male vs. female)	16.2 (6.2; 26.2)	0.0015^**^	0.12
Marital status (in a relationship vs. single)	−118.3 (-26.5; −110.1)	0.0000^***^	−10.17
Work system (two-shift vs. other)	10.4 (2.8; 18.0)	0.0072^**^	0.10
Overtime work (yes vs. no)	9.9 (2.3; 17.5)	0.0106^*^	0.10

^*^, ^**^, ^***^ *p*-values indicate the level of statistical significance.

As Anytime Wakeability increased by 1 point, the level of stress decreased by 1.5 points on average. The same pattern was observed for Daytime Wakeability, whereas each 1-point increase in Anytime Sleepability resulted in a 2-point increase in the overall stress measure. The study found that being male was an independent predictor of higher stress levels. In the multivariate model, men scored on average 16.2 points higher on the stress scale than women. People in a relationship showed lower levels of work-related stress (by 18.2 points on average), while working in a two-shift system and working overtime were associated with an increase in the level of stress by 10.4 and 9.9 points, respectively. The observed relationships should be interpreted as co-occurrence of the analyzed factors with stress, rather than causation (Table 4).

The model explained 16.6% of the variability in the level of occupational stress (R^2^ = 16.6%), which indicates a moderate predictive value of the analyzed variables.

### 3.4 Sick leave vs. the level of work-related stress (Subjective Work Assessment)

The relationship between the sick leave use and the level of work-related stress (as measured by SWA questionnaire) was analyzed. In the analysis, descriptive statistics of the SWA measures were compared against the status of sick leave use. The significance of differences in stress levels between the two groups was assessed using the Mann-Whitney test.

For all stress measures, significantly higher stress levels were observed among nurses who had taken sick leave in the past year. For example, the mean score of overall stress among nurses taking sick leave was 156.5 points, which was over 16 points higher than the mean score among other nurses (140.1 points). The difference was statistically significant (*p* < 0.001).

People who took sick leave were characterized by significantly higher levels of occupational stress compared to those who did not take sick leave (Table 5).

**Table 5:** The relationship between the level of work-related stress and the use of sick leave.

Subjective work assessment	Sick leave during the last year?						*P*
	Yes (*N* = 226)			No (*N* = 362)			
	Mean	Median	Std. dev.	Mean	Median	Std. dev.	
Overall stress	156.5	151	50.4	140.1	135	47.2	0.0001^***^
Mental workload	25.5	25	9.0	22.4	21	8.6	0.0001^***^
Lack of reward	22.9	23	8.1	20.1	19	7.6	0.0001^***^
Organizational uncertainty	20.7	20	7.1	18.8	17	6.8	0.0014^**^
Social interaction	14.1	13	5.2	12.8	11	4.6	0.0030^**^
Sense of threat	14.4	14	4.9	13.5	13	4.7	0.0291^*^
Physical strain	11.3	12	5.3	9.8	8	5.5	0.0006^***^
Unpleasant working conditions	8.4	9	3.7	7.5	6	3.7	0.0053^**^
Lack of control	11.0	10	3.9	10.2	9	3.8	0.0096^**^
Lack of support	8.0	8	3.2	6.7	6	3.0	0.0000^***^
Sense of responsibility	11.5	11	4.1	10.7	10	3.8	0.0288^*^

*p* – test probability value calculated with the Mann-Whitney test. ^*^, ^**^, ^***^ *p*-values indicate the level of statistical significance.

### 3.5 Other factors that have an impact on the use of sick leave

The table below shows the frequency of taking sick leave depending on the selected socio-demographic and professional characteristics of the respondents, such as gender, age, marital status, length of service, education level, nature of work performed, overtime work and having an additional job.

The analysis has shown statistically significant differences in the use of sick leave depending on age (*p* = 0.0072), marital status (*p* = 0.0038), having a second job (*p* = 0.0093), and working overtime (*p* = 0.0048). With respect to age, people under 30 years of age were the least likely to use sick leave (23.4%). In older age groups, this percentage was higher, and remained at a similar level of approximately 43–44% from the age of 41. Furthermore, more frequent use of sick leave was noted among people after the breakup of a relationship (56.2%) compared to those in a relationship (35.6%) or single people (37.4%). Respondents with additional employment were more likely to use sick leave than respondents without a second job (45.5% vs. 34.5%). A similar relationship was observed for overtime work – those working overtime were more likely to use sick leave than those who did not work overtime (43.2% vs. 31.7%) (Table 6).

**Table 6:** Analysis of the relationship between selected socio-demographic variables and the need for taking sick leave.

Characteristics	Group	Sick leaves		*p*
		*N*	%	
Gender	Female	183	37.4%	0.2622
	Male	43	43.4%	
Age	≤ 30 years	25	23.4%	0.0072^**^
	31-40 years	56	38.1%	
	41-50 years	60	44.4%	
	51-60 years	76	43.2%	
	> 60 years	9	39.1%	
Marital status	Single	34	37.4%	0.0038^**^
	In a relationship	151	35.6%	
	Relationship breakdown	41	56.2%	
Education level	Secondary nursing school	38	44.7%	0.4165
	Bachelor's degree	81	38.4%	
	Master's degree	107	36.8%	
Place of employment	Medical ward	78	35.8%	0.4853
	Surgical ward	68	37.4%	
	Intensive care unit	42	44.7%	
	Primary healthcare	38	40.4%	
Work system	One-shift	55	41.0%	0.3736
	Two-shift	128	36.3%	
	Three-shift	43	43.0%	
Additional jobs	Yes	91	45.5%	0.0093^**^
	No	132	34.5%	
Overtime work	Yes	149	43.2%	0.0048^**^
	No	76	31.7%	

*p* – test probability value calculated with the chi-square test of independence. ^**^ *p*-values indicate the level of statistical significance.

### 3.6 Multivariate analysis

A multivariate model illustrating the combined effect of demographic, social and occupational factors, as well as stress levels, on the likelihood of taking sick leave was constructed using logistic regression. In the logistic regression model, the dependent variable is dichotomous, and the model estimates the probability of the defined outcome (in our case, sick leave).

The initial set of independent variables included characteristics such as: age (as a numerical variable), marital status, additional place of employment, overtime work, and overall stress level (as measured by SWA) (also as a numerical variable). These are characteristics which, in a univariate analysis, were found to be statistically significantly associated with the use of sick leave.

Statistically insignificant factors were eliminated using stepwise regression; the final results of the analysis are presented in the model below.

The model did not include two factors which, in earlier univariate analyses, had a statistically significant impact on the use of sick leave-in the multivariate analysis, marital status and working overtime were found to be insignificant. The lack of significance of these factors can be explained by the associations of marital status with age, and working overtime with having a second job.

The analysis has confirmed statistical significance of three factors: age, overall stress, and additional work. A nurse who is one year older has a 2.6% increased chance of taking sick leave, a 1-point increase in stress level increases the chance of taking sick leave by 0.7%, and taking additional work increases the chance of taking sick leave by 55.7%. Obviously, these effects can be combined-for a nurse who is 10 years older, has 20 points higher stress level, and takes on additional work, the chance of taking sick leave increases (1.026)^10^ (1.007)^20^ 1.557, i.e., 2.31 times (in other words, by 131%) (Table 7).

**Table 7:** Results of the logistic regression analysis.

Independent variables	Sick leave (yes vs. no)	
	OR (95% p.u.)	*p*
Age (years)	1.026 (1.011-1.042)	0.0010^***^
Overall stress (score)	1.007 (1.004-1.011)	0.0001^***^
Second job (yes vs. no)	1.557 (1.087-2.231)	0.0158^*^

^*^, ^***^ *p*-values indicate the level of statistical significance

## 4 Discussion

The present study examined the relationships between self-reported sleep–wake characteristics, occupational stress, and sick leave among practicing nurses. The results demonstrated statistically significant associations between selected dimensions of the Sleep–Wake Pattern Assessment Questionnaire (SWPAQ) and the level of perceived occupational stress. Although these associations were statistically significant, their strength was weak, indicating that self-reported sleep–wake characteristics represent only one of several factors related to occupational stress among nurses. Therefore, the observed findings should be interpreted as associations rather than evidence of causal relationships, particularly in view of the cross-sectional design of the study. Nevertheless, the direction of the observed relationships is consistent with previous reports indicating that sleep–wake functioning, shift work, and psychosocial working conditions are closely interrelated among nursing staff (Rosa et al., 2019).

One of the principal findings of the present study was the inverse association between the SWPAQ dimension “Anytime Wakeability” and perceived occupational stress. Nurses reporting a greater self-perceived ability to remain awake in different daily situations tended to report lower occupational stress scores. Since the SWPAQ assesses subjective sleep–wake characteristics rather than physiological circadian functioning, these findings should be interpreted as reflecting self-reported behavioral tendencies rather than objective chronobiological adaptation. Similar associations have been reported in previous studies indicating that more favorable sleep–wake characteristics and better adaptation to shift work are associated with lower levels of perceived stress and fewer sleep-related complaints among nurses (de Bruijn et al., 2025; Fusz et al., 2025; Li et al., 2024). However, the cross-sectional nature of the present study does not allow conclusions regarding the direction of these relationships. It is equally plausible that lower occupational stress is associated with more favorable self-reported sleep–wake functioning, or that both are influenced by other occupational and psychosocial factors.

The present study also demonstrated a positive association between the SWPAQ dimension “Anytime Sleepability” and perceived occupational stress. Nurses who reported a greater self-perceived tendency to fall asleep under different circumstances also reported higher levels of occupational stress. It should be emphasized, however, that the SWPAQ dimension “Anytime Sleepability” reflects a subjective sleep–wake characteristic and should not be interpreted as a direct measure of excessive daytime sleepiness, sleep disorders, fatigue, or impaired physiological regulation of sleep. Therefore, the observed association should be interpreted with caution and considered as a relationship between self-reported sleep–wake characteristics and perceived occupational stress rather than evidence of underlying clinical sleep problems.

Nevertheless, the direction of the observed association is consistent with previous studies suggesting that poorer subjective sleep quality, sleep-related complaints, and difficulties associated with shift work frequently coexist with higher levels of occupational stress among nurses (Slavish et al., 2022; Rocha and Martino, 2010; Costa et al., 2014; Gu et al., 2025). Similarly, Gu et al. (2025) reported a high prevalence of excessive daytime sleepiness among nurses worldwide, while Zhang et al. (2023) highlighted that shorter sleep duration and poorer sleep quality were associated with poorer wellbeing and higher perceived stress. Owen and Lach (2025) also emphasized that sleep-related problems are common among shift-working nurses, whereas Serafin et al. (2021) demonstrated that higher stress levels were associated with insomnia symptoms and less adaptive coping strategies. Although these findings provide an important context for interpreting our results, it should be noted that the present study did not directly assess daytime sleepiness, insomnia, or sleep quality. Consequently, these mechanisms should be regarded as possible explanations rather than conclusions supported by the collected data.

Another important finding of the present study was the association between selected self-reported sleep–wake characteristics and the use of sick leave. Nurses reporting less favorable scores in selected SWPAQ dimensions were more likely to report sick leave during the analyzed period. Although these findings suggest that sleep–wake characteristics may be related to work functioning, they should not be interpreted as evidence that these characteristics directly influence sickness absence. Sick leave is a multifactorial outcome affected by individual health status, working conditions, psychosocial factors, organizational policies, and family circumstances. Therefore, the observed associations most likely reflect the combined influence of multiple occupational and personal determinants.

Previous studies have similarly demonstrated that sleep-related problems, high occupational stress, and demanding shift schedules are associated with increased sickness absence and reduced work ability among healthcare professionals (Gohar et al., 2020; Cho and Steege, 2021; Lim et al., 2026; Okuhara et al., 2021). These findings provide a broader context for interpreting the present results; however, because the current study did not examine the reasons for sick leave or participants' clinical health status, no conclusions can be drawn regarding the mechanisms underlying these associations. Further longitudinal studies are needed to clarify whether self-reported sleep–wake characteristics predict future sickness absence or simply coexist with other factors contributing to absenteeism.

The present study also identified several sex-related differences in selected SWPAQ dimensions. Female nurses reported significantly higher scores for Anytime Sleepability, whereas male nurses obtained higher scores for Anytime Wakeability and Morning Lateness. These findings indicate differences in self-reported sleep–wake characteristics between women and men; however, the underlying reasons remain unclear. They may reflect differences in work organization, domestic responsibilities, recovery opportunities, or other biological and psychosocial factors that were not assessed in the present study. Therefore, these findings should be interpreted cautiously.

Previous studies have reported sex differences in sleep patterns, adaptation to shift work, and perceived occupational stress among healthcare professionals (Lim et al., 2026; Okuhara et al., 2021; Blackley et al., 2019). Women are generally more likely to report sleep-related complaints and poorer subjective sleep quality, whereas men often demonstrate different patterns of adaptation to irregular work schedules. Nevertheless, findings across studies remain inconsistent, suggesting that sex alone is unlikely to explain these differences. Occupational characteristics, family responsibilities, and lifestyle factors may also contribute to the observed variability (Lim et al., 2026; Okuhara et al., 2021; Blackley et al., 2019). Future studies should examine these factors in greater detail to better understand the mechanisms underlying sex differences in self-reported sleep–wake characteristics.

A literature review by Okuhara et al. (2021), which included 132 studies published over the previous decade, identified numerous occupational, organizational, and psychological factors associated with stress among both male and female nurses. Although some studies reported sex-related differences in occupational stress, others found no significant associations, indicating that the available evidence remains inconsistent. Psychological and work-related factors were the most frequently identified determinants of stress across the reviewed studies.

Our findings should be interpreted within this broader context. Although male nurses in the present study reported higher levels of occupational stress, the cross-sectional design does not allow conclusions regarding the reasons for this difference. It is possible that sex-related differences coexist with other occupational, organizational, or psychosocial factors that were not assessed in the present study. Similar observations have been discussed by Blackley et al. (2019), who highlighted the unique professional experiences and workplace challenges encountered by men working in the nursing profession.

## 5 Conclusions

The present study demonstrated significant associations between selected self-reported sleep–wake characteristics, occupational stress, and sick leave among practicing nurses. Although these associations were generally weak, they suggest that subjective sleep–wake functioning may represent one of several factors related to occupational wellbeing in this professional group.

The findings also indicate sex-related differences in selected sleep–wake characteristics and occupational stress; however, the underlying mechanisms remain unclear and require further investigation.

Given the cross-sectional design and the use of self-report measures, the observed relationships should be interpreted with caution and should not be considered evidence of causal associations. Future longitudinal studies incorporating objective measures of sleep and work-related factors are needed to better understand the relationships between sleep–wake characteristics, occupational stress, and sickness absence among nurses.

## 6 Practical implications

The findings of the present study suggest that self-reported sleep–wake characteristics and occupational stress are interrelated among practicing nurses. From a practical perspective, regular assessment of occupational stress and monitoring nurses' wellbeing may help identify staff members who could benefit from preventive and supportive interventions.

Healthcare organizations should promote evidence-based strategies aimed at reducing occupational stress, including organizational support, stress management programmes, and interventions designed to improve working conditions and recovery opportunities. Such initiatives may contribute to improved occupational wellbeing and work functioning among nurses.

The observed associations also highlight the importance of considering individual differences in self-reported sleep–wake characteristics when organizing shift work. Although the present study does not provide evidence of causal relationships, recognizing these individual differences may support more personalized approaches to workforce management and occupational health promotion. Future studies should evaluate whether integrating information on sleep–wake characteristics into work organization translates into measurable improvements in nurses' wellbeing and work-related outcomes.

### 6.1 Limitations of the study

The present study has several limitations that should be considered when interpreting the findings. First, the cross-sectional design precludes conclusions regarding causal relationships between self-reported sleep–wake characteristics, occupational stress, and sick leave. The observed associations should therefore be interpreted as correlational rather than causal.

Second, although the study included a relatively large sample of practicing nurses, the observed associations were generally weak, suggesting that self-reported sleep–wake characteristics represent only one of several factors related to occupational stress. Other organizational, psychosocial, health-related, and individual factors that were not assessed in the present study may also have contributed to the observed relationships.

Third, all variables were assessed using self-report questionnaires, which may be subject to reporting bias and shared method variance. In addition, the study did not include objective measures of sleep or circadian functioning, nor did it assess the specific reasons for sickness absence.

Finally, the characteristics of the study sample may limit the generalisability of the findings to other groups of nurses working in different healthcare systems or organizational settings. Future longitudinal studies combining self-reported and objective measures of sleep–wake functioning, together with broader assessments of occupational and psychosocial factors, are warranted to better understand the relationships identified in the present study.

## Data Availability

The original contributions presented in the study are included in the article/supplementary material, further inquiries can be directed to the corresponding author.
